# Too uncertain to consent, too supportive to refuse: the sociocultural dilemma of hesitant organ donors in Kazakhstan

**DOI:** 10.3389/fpubh.2025.1602008

**Published:** 2025-05-19

**Authors:** Aidos Bolatov, Aruzhan Asanova, Aigerim Abdiorazova, Yuriy Pya

**Affiliations:** ^1^University Medical Center Corporate Fund, Astana, Kazakhstan; ^2^Shenzhen University Medical School, Shenzhen University, Shenzhen, China; ^3^School of Medicine, Astana Medical University, Astana, Kazakhstan

**Keywords:** organ donation, hesitancy, barriers, public attitudes, health policy

## Abstract

**Background:**

Understanding the factors influencing posthumous organ donation decisions is essential for developing effective strategies to increase donor registration. While previous studies have explored reasons for consent and refusal, less attention has been given to individuals who defer the decision to their families (Decision Left to Close Relatives, DLCR). This study examines the sociodemographic, institutional, and cultural factors influencing donation preferences, with a focus on the DLCR group as a transitional category between consent (LC) and refusal (LR).

**Methods:**

A cross-sectional survey was conducted among 1,333 participants in Kazakhstan. Donation preferences were categorized into Lifetime Consent (35.3%), Lifetime Refusal (21.4%), and DLCR (43.4%). Participants completed measures assessing knowledge, attitudes, and perceived barriers to organ donation. Principal Component Analysis (PSA) identified two key dimensions of perceived barriers: institutional and cultural barriers. Linear regression and mediation analyses were performed to examine predictors of attitudes toward organ donation.

**Results:**

The DLCR group held intermediate attitudes toward donation, significantly higher than LR but lower than LC (*p* < 0.001), moreover, 44.4% of the DLCR group had a favorable attitude toward organ donation. A critical finding was the high level of uncertainty about how to declare donation status among DLCR participants, significantly higher than in both LC and LR (*p* < 0.05). PCA revealed that DLCR individuals were institutionally closer to LC but culturally aligned with LR, suggesting that cultural concerns are the stronger barrier preventing proactive consent. Among DLCR participants, knowledge positively predicted donation attitudes (β = 0.223, *p* < 0.001), while cultural and religious barriers had the strongest negative effect (β = −0.290, *p* < 0.001). Language preference also emerged as a factor, with Russian speakers demonstrating significantly more favorable attitudes than Kazakh speakers. Specialization (medical vs. non-medical) had no direct effect on donation attitudes (*p* = 0.777), but it influenced attitudes indirectly through institutional (β = −0.223, p < 0.001) and cultural barriers (β = 0.194, *p* = 0.003).

**Conclusion:**

Both procedural uncertainty and cultural-religious factors influence the hesitation of DLCR individuals to commit to donation, with cultural concerns having a stronger effect. Language preference also shapes attitudes, reflecting broader sociocultural framings. Reducing uncertainty and addressing cultural misconceptions, particularly among the DLCR group, may be key to increasing donor registration.

## 1 Introduction

Organ donation remains one of the most effective ways to save lives worldwide for people with an end-stage organ failure. Despite the clear potential to save lives, there remains a significant gap between the demand for organs and their availability. According to the World Health Organization (WHO), every year, millions of people worldwide die while waiting for an organ transplant (1). In fact, the number of people requiring an organ transplant is steadily increasing, yet the number of available organs fails to meet this growing demand. This disparity highlights the need for more individuals to consider posthumous organ donation, as well as for a better understanding of the factors that influence people’s decisions on the matter.

The decision to donate organs after death is deeply personal. It involves a complex interplay of sociodemographic, cultural, psychological, and institutional factors making it not only an individual choice but also one that often extends to families and societies (2). The reluctance to make a lifetime consent or refusal regarding posthumous organ donation is often referred as hesitancy, which ultimately results in decisions being made by family members during critical moments. While much research has focused on the reasons people consent or refuse posthumous organ donation, less attention has been given to those who hesitate to make a decision and defer it to their families (3). This group—identified in this study as the Decision Left to Close Relatives (DLCR) group—represents a significant portion of the population, yet little is known about the underlying reasons for deferring this choice.

Organ donation rates vary significantly across the world and are commonly measured by the number of actual deceased donors per million population (pmp). The United States, operating under an opt-in system, has relatively efficient donation programs, ranking among the top performers globally, but still faces challenges in converting potential donors into actual donors (4). Spain leads worldwide in organ donation, with posthumous organ donation rates of 33–35 donors pmp, attributed to its presumed consent system (opt-out), strong transplant coordination, and public trust (5). France, with 23.2 donors pmp, also benefits from an opt-out system and donations after brain and circulatory death, yet it still lags behind Spain due to efficiency and trust issues (6). Across the European Union, donation rates vary widely, with countries like Spain, Portugal, and Austria leading, while legislative and cultural differences hinder a unified opt-out approach (5). Despite efforts to increase posthumous organ donation, Kazakhstan remains significantly below global standards, with 0.3 deceased donors pmp in 2024, highlighting the need for improved public awareness and policy interventions (7).

At the same time, Kazakhstan’s healthcare system relies primarily on living donors. Between 2012 and 2024, Kazakhstan performed a total of 2,753 organ transplants, with 83.9% from living donors and 16.1% from deceased donors. Overall, 121 deceased donors have been recorded from 2012 to the present day. As of September 2024, more than 4,000 individuals in Kazakhstan were on the waiting list for posthumous organ donation, including 102 children (7). In this way, the question of how to encourage more individuals to make their preferences known regarding posthumous organ donation is critical, especially as it could help to reduce the waiting times and potentially save more lives.

Kazakhstan’s approach to organ donation follows an opt-in system, meaning that individuals must actively consent to posthumous organ donation by either registering their decision at a primary healthcare facility or through the electronic government portal (8). If a person has not made a lifetime decision, and brain death is confirmed, the deceased’s family decides on organ donation on their behalf. As of 2024, only 111.6 thousand out of 20.33 million people in Kazakhstan had recorded their decision on posthumous organ donation (9). Among them, 104.4 thousand opted out, while 7.2 thousand gave their consent (9). In this way, families play a vital role in organ donation, as their objections to organ procurement are a major reason why organs are not retrieved in many countries (10).

Research suggests that individuals may hesitate to make a definitive decision due to a lack of knowledge, fear of potential medical errors, or trust concerns surrounding the posthumous organ donation process (11, 12). Furthermore, socio-cultural and family dynamics can heavily influence this deferral, with many individuals relying on their family members to make the decision in their stead (13). Prior studies indicate that sociodemographic factors such as age, gender, education, and socioeconomic status play crucial roles in shaping one’s attitude toward organ donation (14). For example, younger individuals tend to show more favorable attitudes toward organ donation than older individuals, and those with higher education levels are more likely to support organ donation (15–17). Additionally, religious beliefs often dictate one’s stance on organ donation, with certain faiths expressing more opposition to organ donation than others. Psychological factors such as fear of bodily harm, concerns over the sanctity of the body after death, and perceptions of medical mistrust also contribute to the reluctance to make a decision or lifetime refusal (14).

This study aims to explore these factors in greater depth to determine whether institutional concerns, such as procedural fairness and trust in medical systems, or cultural concerns, such as religious and societal norms, have a more significant influence on individuals’ hesitancy to make decisions about organ donation. By focusing specifically on the DLCR group—those individuals who defer their decision to their families—this research seeks to uncover the reasons behind this widespread reluctance to make lifetime decisions on organ donation. The study will examine how sociodemographic, institutional, and cultural factors intersect and influence individuals’ decisions, providing valuable insights into the barriers that prevent people from expressing their preferences for organ donation during their lifetime. Understanding these factors is critical to developing effective strategies that encourage individuals to express their preferences for posthumous organ donation while alive, potentially addressing the issue of the shortage of transplantable organs.

## 2 Methods

This cross-sectional study aimed to explore factors influencing posthumous organ donation decisions, particularly among individuals who defer the decision to their families (Decision Left to Close Relatives, DLCR group), compared to those who provide Lifetime Consent (LC) or express Lifetime Refusal (LR).

### 2.1 Study setting

This study was conducted in the Republic of Kazakhstan, a Central Asian country with a population of approximately 20.3 million as of 2025. The country is ethnically diverse and operates under a mixed public-private healthcare system. Organ donation in Kazakhstan follows an opt-in model, requiring individuals to register their consent through official channels. As the study aimed to assess national attitudes, data were collected from a broad and diverse sample across the country, without restriction to specific regions or cities.

### 2.2 Data collection

Participants completed a structured questionnaire assessing sociodemographic characteristics, knowledge, attitudes, and perceived barriers toward posthumous organ donation. Collected sociodemographic variables included age, gender, ethnicity, language preference, education, occupation, family status, number of children, living area (urban/rural), economic well-being, and religious affiliation. The survey was administered in both Kazakh and Russian to ensure accessibility and comprehension for participants from different linguistic backgrounds. The survey also contained validated scales measuring knowledge and perceived barriers to organ donation.

### 2.3 Study participants

A total of 1,333 participants were recruited using an online survey distributed through various social media platforms, including Facebook, Instagram, and WhatsApp. The survey was open to adults aged 18 years and older residing in Kazakhstan, and participation was voluntary and anonymous. The final sample included 396 non-medical professionals and 937 individuals with a medical background.

Due to the online nature of recruitment, the sample was skewed toward younger, urban, more educated, and digitally connected individuals. Women, people with medical training, married participants, and residents of urban areas were particularly overrepresented. Consequently, individuals from lower socioeconomic backgrounds, rural regions, and those with limited digital access or lower educational attainment may be underrepresented in this dataset. While the large sample enhances analytical robustness, caution is warranted when generalizing these findings to the broader population.

### 2.4 Measures

The questionnaire included items to collect information on key sociodemographic variables: gender, age, ethnicity, native language, occupation, education, marital/family status, presence of children, type of living area (urban or rural), and religious affiliation. These variables were used to assess potential demographic influences on participants’ preferences regarding posthumous organ donation.

Religiosity was measured using a single-item self-assessment: “How religious do you consider yourself to be?” Responses were recorded on a five-point Likert scale ranging from 1 (not religious at all) to 5 (very religious), with higher scores indicating greater perceived religiosity.

To assess perceived financial security, participants were asked: “How comfortable do you feel in your current financial situation?” Responses were rated on a five-point scale, where 1 represented “very uncomfortable” and 5 represented “very comfortable.”

Participants’ knowledge of organ donation procedures, eligibility, and benefits was assessed using a previously published seven-item scale (18). The items covered procedural aspects, eligibility criteria, and benefits of posthumous organ donation. Each item was presented in a True/False/I do not know format. Correct answers received 1 point, while incorrect or “I do not know” responses received 0. The total score ranged from 0 to 7, with higher scores indicating greater knowledge and awareness of organ donation.

Participants’ perceptions of barriers to organ donation were evaluated using previously validated nine statements, each rated on a 5-point Likert scale (1 = strongly disagree, 5 = strongly agree) (18). The barriers assessed included concerns about medical complications, opposition from family members, religious and cultural beliefs, trust in the medical system, potential for unethical practices such as organ trafficking, fear of reduced medical care, discomfort with posthumous body use, insufficient awareness and education, and financial concerns. In addition to these nine established barriers, this study introduced a new barrier: “Lack of knowledge on how to declare an organ donation decision,” addressing procedural uncertainties that may influence individuals’ willingness to commit to posthumous donation.

Attitudes toward organ donation were measured using the statement: “I have a positive attitude toward donating my organs after death,” rated on a 5-point Likert scale (1 = strongly disagree, 5 = strongly agree). Higher scores indicated more favorable attitudes toward posthumous donation.

Participants’ preferences regarding posthumous organ donation were assessed using the question: “How would you like to express your decision regarding posthumous organ donation?” Response options included: lifetime consent (LC), lifetime refusal (LR), and decision left to close relatives (DLCR). These categories were used to classify participants into three decision groups for further analysis.

### 2.5 Statistical analysis

Descriptive analyses were conducted to summarize participant characteristics, knowledge levels, and perceived barriers across the three organ donation decision groups (LR, DLCR, LC). Means and standard deviations were reported for continuous variables, while categorical variables were summarized using frequencies and percentages. To compare differences in knowledge, attitudes, and perceived barriers among the three decision groups (LR, DLCR, LC), One-Way ANOVA was performed. This method assessed whether the mean values of each variable differed significantly across groups. Tukey’s post-hoc tests were conducted for pairwise comparisons, identifying which groups significantly differed from each other. To evaluate whether language remained an independent predictor of organ donation preferences after accounting for potential confounders, a series of multinomial logistic regression analyses were conducted. To explore the underlying structure of perceived barriers, Principal Component Analysis (PCA) with Varimax rotation was conducted. The goal was to reduce the number of variables and identify latent components that explain the variance in perceived barriers. The Kaiser-Meyer-Olkin (KMO) measure of sampling adequacy and Bartlett’s test of sphericity were used to assess the suitability of the data for factor analysis. To explore predictors of attitudes toward posthumous organ donation within the DLCR group, a linear regression model was applied. The dependent variable was attitude toward organ donation, while independent variables included sociodemographic factors, knowledge levels and perceived barriers. Standardized beta coefficients (β) were used to determine the relative strength of each predictor. The model’s goodness-of-fit was evaluated using R^2^ values. A mediation analysis was conducted to examine whether different barriers mediate the relationship between specialization (medical vs. non-medical background) and attitudes toward posthumous organ donation within the DLCR group. All statistical analyses were conducted using Jamovi software (version 2.6.17), with a significance threshold set at *p* < 0.05.

## 3 Results

A total of 1,333 participants were included in the study (Table 1). The sample had a mean age of 36.7 years (SD = 11.5, range: 18–70). The majority of participants were female (78.2%), Kazakh (81.5%), and predominantly from urban areas (79.2%). Regarding language preference, Kazakh speakers accounted for 50.6% of the sample, while Russian speakers comprised 46.4%. Religious affiliation varied, with 72.2% identifying as Muslim, 9.8% as atheists, 9.2% as agnostics, and 7.5% as Christian. Educational attainment was relatively high: 65.6% of participants held an undergraduate degree, 17.9% had special-professional education, 8.4% had completed postgraduate studies, while smaller proportions had finished high school (7.2%) or only middle school (0.9%). Medical professionals reported higher levels of religiosity (2.77 ± 1.29) compared to non-medical participants (2.35 ± 1.18). Employment status also differed, with 83.0% of medical professionals being employed compared to 62.9% in the non-medical group.

**Table 1:** Socio-demographic characteristics of study population (*N* = 1,333).

Variable	Non-Medical (*n* = 396)	Medical (*n* = 937)
Gender		
Male (*n* = 291, 21.8%)	96 (24.2%)	195 (20.8%)
Female (*n* = 1,042, 78.2%)	300 (75.8%)	742 (79.2%)
Age group		
18–24 years (*n* = 168, 12.6%)	70 (17.7%)	98 (10.5%)
25–34 years (*n* = 504, 37.8%)	155 (39.1)	349 (37.2%)
35–44 years (*n* = 323, 24.2%)	108 (27.3%)	215 (22.9%)
>45 years (*n* = 338, 25.4%)	63 (15.9%)	275 (29.3%)
Ethnicity		
Kazakh (*n* = 1,086, 81.5%)	322 (81.3%)	764 (81.5%)
Russian (*n* = 117, 8.8%)	50 (12.6%)	67 (7.2%)
Other (*n* = 130, 9.8%)	24 (6.1%)	106 (11.3%)
Language		
Kazakh (*n* = 675, 50.6%)	119 (30.1%)	556 (59.3%)
Russian (*n* = 619, 46.4%)	265 (66.9%)	354 (37.8%)
Other (*n* = 39, 2.9%)	12 (3.0%)	27 (2.9%)
Occupation		
Student (*n* = 168, 12.6%)	48 (12.1%)	120 (12.8%)
Employed (*n* = 1,027, 77.0%)	249 (62.9%)	778 (83.0%)
Self-employed (*n* = 78, 5.9%)	62 (15.7%)	16 (1.7%)
Unemployed (*n* = 36, 2.7%)	30 (7.6%)	6 (0.6%)
Pensioner (*n* = 24, 1.8%)	7 (1.8%)	17 (1.8%)
Educational level		
Middle-school (*n* = 12, 0.9%)	1 (0.3%)	11 (1.2%)
High-school (*n* = 96, 7.2%)	25 (6.3%)	71 (7.6%)
Special-professional education (*n* = 239, 17.9%)	27 (6.8%)	212 (22.6%)
Undergraduate (*n* = 874, 65.6%)	318 (80.3%)	556 (59.3%)
Post-graduate (*n* = 112, 8.4%)	25 (6.3%)	87 (9.3%)
Family status		
Single (*n* = 386, 29.0%)	157 (39.6%)	229 (24.4%)
Married (*n* = 790, 59.3%)	192 (48.5%)	598 (63.8%)
Divorced (*n* = 120, 9.0%)	39 (9.8%)	81 (8.6%)
Widow (*n* = 37, 2.8%)	8 (2.0%)	29 (3.1%)
Children		
No (*n* = 451, 33.8%)	187 (47.2%)	264 (28.2%)
Yes (*n* = 882, 66.2%)	209 (52.8%)	673 (71.8%)
Living area		
Rural (*n* = 277, 20.8%)	44 (11.1%)	233 (24.9%)
Urban (*n* = 1,056, 79.2%)	352 (88.9%)	704 (75.1%)
Religion affiliation		
Islam (*n* = 962, 72.2%)	217 (54.8%)	745 (79.5%)
Christian (*n* = 100, 7.5%)	40 (10.1%)	60 (6.4%)
Agnosticism (*n* = 122, 9.2%)	61 (15.4%)	61 (6.5%)
Atheism (*n* = 130, 9.8%)	70 (17.7%)	60 (6.4%)
Other (*n* = 19, 1.4%)	8 (2.0%)	11 (1.2%)
Religiosity (M = 2.65, SD = 1.27, 1–5)	2.35 ± 1.18	2.77 ± 1.29

Regarding their preference for posthumous organ donation, 43.4% (*n* = 578) preferred to leave the decision to their close relatives (Decision Left to Close Relatives group, DLCR), while 21.4% (*n* = 285) expressed lifetime refusal (LR), and 35.3% (*n* = 470) gave lifetime consent (LC). The analysis focused on the DLCR group, comparing it with the LR and LC groups across key sociodemographic, religious, and attitudinal factors (Table 2).

**Table 2:** Sociodemographic, religious, and knowledge-based factors associated with preference for decision on posthumous organ donation (*N* = 1,333).

Variable	Preference for decision on posthumous organ donation			χ^2^/F, *p* Post-hoc
	Lifetime refusal (*n* = 285)	Decision left to close relatives (*n* = 578)	Lifetime consent (*n* = 470)	
Gender				
Male	74 (25.4%)	121 (41.6%)	96 (33.0%)	3.7, *p* = 0.160
Female	211 (20.2%)	457 (43.9%)	374 (35.9%)	
Age group				
18–24 years	24 (14.3%)	60 (35.7%)	84 (50.0%)	92.0, *p* < 0.001
25–34 years	83 (16.5%)	201 (39.9%)	220 (43.7%)	
35–44 years	72 (22.3%)	139 (43.0%)	112 (34.7%)	
>45 years	106 (31.4%)	178 (52.7%)	54 (16.0%)	
Ethnicity				
Kazakh	222 (20.4%)	473 (43.6%)	391 (36.0%)	4.0, *p* = 0.410
Russian	28 (23.9%)	49 (41.9%)	40 (34.2%)	
Other	35 (26.9%)	56 (43.1%)	39 (30.0%)	
Language				
Kazakh	180 (26.7%)	338 (50.1%)	157 (23.3%)	89.8, *p* < 0.001
Russian	102 (16.5%)	225 (36.3%)	292 (47.2%)	
Other	3 (7.7%)	15 (38.5%)	21 (53.8%)	
Occupation				
Student	15 (8.9%)	53 (31.5%)	100 (59.5%)	69.2, *p* < 0.001
Employed	251 (24.4%)	463 (45.1%)	313 (30.5%)	
Self-employed	11 (14.1%)	30 (38.5%)	37 (47.4%)	
Unemployed	4 (11.1%)	17 (47.2%)	15 (41.7%)	
Pensioner	4 (16.7%)	15 (62.5%)	5 (20.8%)	
Educational level				
Middle-school	3 (25.0%)	6 (50.0%)	3 (25.0%)	60.7, *p* < 0.001
High-school	26 (27.1%)	46 (47.9)	24 (25.0%)	
Special-professional education	73 (30.5%)	127 (53.1%)	39 (16.3%)	
Undergraduate	160 (18.3%)	360 (41.2%)	354 (40.5%)	
Post-graduate	23 (20.5%)	39 (34.8%)	50 (44.6%)	
Specialization				
Non-medical	68 (17.2%)	144 (36.4%)	184 (46.5%)	31.1, *p* < 0.001
Medical	217 (23.2%)	434 (46.3%)	286 (30.5%)	
Family status				
Single	58 (15.0%)	132 (34.2%)	196 (50.8%)	60.8, *p* < 0.001
Married	191 (24.2%)	375 (47.5%)	224 (28.4%)	
Divorced	26 (21.7%)	52 (43.3%)	42 (35.0%)	
Widow	10 (27.0%)	19 (51.4%)	8 (21.6%)	
Children				
No	62 (13.7%)	143 (31.7%)	246 (54.5%)	112.0, *p* < 0.001
Yes	223 (25.3%)	435 (49.3%)	224 (25.4%)	
Living area				
Rural	68 (24.5%)	147 (53.1%)	62 (22.4%)	25.7, *p* < 0.001
Urban	217 (20.5)	431 (40.8%)	408 (38.6%)	
Religion affiliation				
Islam	226 (23.5%)	460 (47.8%)	276 (28.7%)	101.0, *p* < 0.001
Christian	25 (25.0%)	45 (45.0%)	30 (30.0%)	
Agnosticism	9 (7.4%)	32 (26.2%)	81 (66.4%)	
Atheism	21 (16.2%)	36 (27.7%)	73 (56.2%)	
Other	4 (21.1%)	5 (26.3%)	10 (52.6%)	
Religiosity (1–5)	2.84 ± 1.39	2.79 ± 1.24	2.36 ± 1.18	20.3, *p* < 0.001 LR/DLCR vs. LC, *p* < 0.001
Economic well-being (1–5)	3.14 ± 1.05	3.29 ± 0.95	3.25 ± 1.01	2.2, *p* = 0.116
Knowledge on organ donation (0–7)	3.76 ± 1.93	4.44 ± 1.83	5.63 ± 1.26	145.0, *p* < 0.001 All post-hoc at *p* < 0.001
Posthumous organ donation attitudes (1–5)	2.13 ± 1.11	3.25 ± 1.15	4.48 ± 0.90	504.0, *p* < 0.001 All post-hoc at *p* < 0.001

Age showed a significant effect on donation preferences (*F* = 60.5, *p* < 0.001). The LC group (32.6 ± 9.32) was significantly younger than both the DLCR (38.5 ± 11.97) and LR (40.0 ± 11.68) groups, while no significant difference was found between the LR and DLCR groups. In addition to mean age comparisons, age groups were analyzed using χ^2^-square tests. The association between age group and donation preference was also statistically significant (*p* < 0.001). Younger participants were more likely to express lifetime consent, while older individuals tended to defer the decision or refuse donation. Among those aged 18–24, 50.0% reported lifetime consent, the highest among all age groups, while only 14.3% refused and 35.7% left the decision to relatives. Conversely, participants over 45 years were the most likely to defer (52.7%) or refuse (31.4%) and least likely to consent (16.0%). This trend highlights the greater openness to organ donation among younger individuals and higher levels of hesitancy or opposition among older participants.

Gender distribution was not significantly different across the three groups (*p* = 0.160). Similarly, ethnicity did not show a significant association with donation preferences (*p* = 0.410). However, language was significantly related to decision-making (*p* < 0.001), with Kazakh-speaking participants more likely to be in the DLCR group (50.1%), whereas Russian speakers were more represented in the LC group (47.2%). Multinomial logistic regression analyses were conducted to assess whether language remained an independent predictor of posthumous organ donation preferences after adjusting for key sociodemographic variables (Supplementary Table 1). Across all models, language remained a statistically significant predictor of lifetime consent compared to lifetime refusal. In contrast, language was not a significant predictor of belonging to the DLCR group in any of the models, suggesting that language differences are specifically associated with active consent rather than general indecision or deferral. These findings confirm that language independently influences donation preferences and is not merely a proxy for other sociodemographic characteristics such as education, occupation, or religiosity.

Occupation had a significant influence (*p* < 0.001), with students and self-employed individuals being more likely to opt for lifetime consent, while employed, pensioners and unemployed participants were more likely to leave the decision to their family. Those with medical background were more likely to leave the decision to their families (46.3%) compared to non-medical participants (36.4%). Conversely, individuals without a medical background were more likely to opt for lifetime consent (46.5%) compared to medical professionals (30.5%) (*p* < 0.001).

Education level was significantly associated with preferences for posthumous organ donation (*p* < 0.001). Participants with special-professional education were the most likely to defer the decision to family members (53.1%), whereas those with undergraduate (40.5%) and postgraduate (44.6%) education more frequently reported lifetime consent. In contrast, individuals with middle-school and high-school education were more likely to express lifetime refusal (25.0 and 27.1%, respectively), and only 25.0% of these groups indicated willingness to donate. These findings suggest that higher levels of education are associated with a greater likelihood of actively consenting to organ donation, while lower educational attainment is more often linked to refusal or deferral of decision-making.

Marital status was significantly associated with donation preferences (*p* < 0.001). While single participants were more likely to opt for lifetime consent (50.8%), those in the DLCR group were predominantly married (47.5%), divorced (43.3%) or widow (51.4%). Having children had a strong effect (*p* < 0.001). Parents were significantly more likely to leave the decision to their families (49.3%) compared to non-parents, who had the highest proportion of lifetime consent (54.5%).

Religious affiliation was a key determinant of decision-making (*p* < 0.001). Participants identifying as agnostic (66.4%) and atheist (56.2%) were significantly more likely to opt for lifetime consent compared to Muslim (47.8%) and Christian (45.0%) respondents, who predominantly left the decision to their families. LC participants had significantly lower religiosity scores than both the LR and DLCR groups, with no significant difference between the latter two.

Living area was significantly associated with donation preference (*p* < 0.001). Urban participants were more likely to leave the decision to their families (40.8%) or to opt for lifetime consent (38.6%), whereas rural participants were overrepresented in the DLCR group (53.1%) and less likely to opt for lifetime consent (22.4%). Economic well-being did not show a statistically significant difference across groups (*p* = 0.116), suggesting that financial status was not a key determinant of organ donation preferences.

Knowledge about organ donation was significantly higher in the LC group compared to both the DLCR and LR groups (*p* < 0.001). No significant difference was found between LR and DLCR groups, indicating that individuals hesitant to make a personal commitment to donation tend to have lower awareness of organ donation. Attitudes toward organ donation showed a similar trend (*p* < 0.001). Participants in the LC group had the most positive attitudes, while those in the DLCR group held intermediate views, significantly higher than those who refused posthumous donation.

### 3.1 Perceived barriers to posthumous organ donation

Participants who left the decision about posthumous organ donation to their families (DLCR group) exhibited an intermediate level of concern across most perceived barriers, often aligning more closely with the Lifetime Refusal (LR) group rather than the Lifetime Consent (LC) group (Table 3). Barriers were rated on a 5-point Likert scale ranging from 1 (“strongly disagree”) to 5 (“strongly agree”), with higher scores indicating stronger agreement with the barrier.

**Table 3:** Perceived barriers to posthumous organ donation across decision groups (*N* = 1,333).

Barriers	Preference for decision on posthumous organ donation			F, *p* *Post-hoc*
	Lifetime refusal (*n* = 285)	Decision left to close relatives (*n* = 578)	Lifetime consent (*n* = 470)	
Concerns about potential medical complications arising from organ donation	3.27 ± 1.13	3.58 ± 0.88	3.54 ± 0.99	8.6, *p* < 0.001 LR vs. DLCR/LC, *p* < 0.05
Opposition to organ donation from family members	3.23 ± 1.04	3.10 ± 0.88	2.78 ± 1.03	20.6, *p* > 0.001 LR/DLCR vs. LC, *p* < 0.001
Concerns about the impact of organ donation on cultural or religious beliefs	2.93 ± 1.12	2.80 ± 1.01	1.90 ± 0.99	134.0, *p* < 0.001 LR/DLCR vs. LC, *p* < 0.001
Lack of trust in the medical system’s handling of organ donation	3.41 ± 1.18	3.58 ± 0.97	3.36 ± 1.18	5.9, *p* = 0.003 DLCR vs. LC, *p* < 0.05
Concerns about the potential for organ trafficking or unethical practices	3.55 ± 1.20	3.71 ± 1.03	3.44 ± 1.32	6.5, *p* = 0.002 DLCR vs. LC, *p* < 0.05
Fear that expressing a willingness to become an organ donor may lead to a reduced effort by medical professionals to save the donor’s life	3.23 ± 1.28	3.31 ± 1.10	2.51 ± 1.33	57.2, *p* < 0.001 LR/DLCR vs. LC, *p* < 0.001
Discomfort with the idea of the body being used for organ transplantation posthumously	3.34 ± 1.34	2.85 ± 1.06	1.89 ± 1.05	166.5, *p* < 0.001 All post-hoc at *p* < 0.001
Insufficient awareness and education about organ donation in Kazakhstan	3.55 ± 1.10	3.72 ± 0.99	4.08 ± 1.01	26.4, *p* < 0.001 LR/DLCR vs. LC, *p* < 0.001
Concerns about the financial implications associated with organ donation	3.15 ± 1.12	3.15 ± 0.94	2.81 ± 1.09	15.8, *p* < 0.001 LR/DLCR vs. LC, *p* < 0.001
Lack of knowledge on how to declare organ donation decision	2.38 ± 1.14	2.89 ± 0.97	2.64 ± 1.26	22.3, *p* < 0.001 All post-hoc at *p* < 0.05
Total	32.0 ± 7.91	32.7 ± 5.42	29.0 ± 6.51	50.2, *p* < 0.001 LR/DLCR vs. LC, *p* < 0.001

Values represent mean scores ± standard deviation. Barriers were rated on a 5-point Likert scale (1 = strongly disagree, 5 = strongly agree).

Concerns about medical and ethical issues played a significant role in decision-making. Participants in the DLCR group reported higher concerns about potential medical complications from organ donation compared to those who outright refused donation (*p* < 0.05) but showed no significant difference from the LC group. Lack of trust in how the medical system handles organ donation was also significantly more prevalent in the DLCR group compared to the LC group (*p* < 0.05), while no difference was observed between DLCR and LR. Similarly, fear that expressing willingness to donate might result in reduced medical effort was significantly higher in both the DLCR and LR groups compared to LC (*p* < 0.001). Moreover, concerns about organ trafficking or unethical practices were more pronounced in the DLCR group than in the LC group.

Social and cultural influences were significant determinants of decision-making. Opposition to organ donation from family members was significantly greater in the DLCR and LR groups compared to the LC group (*p* < 0.001), suggesting that family influence played a crucial role in hesitation. Similarly, cultural and religious concerns were more pronounced in both the DLCR and LR groups compared to the LC group (*p* < 0.001), though no significant difference was observed between the first two. Discomfort with the idea of posthumous organ transplantation was also significantly higher in the DLCR group than in the LC group (*p* < 0.001), but lower than in the LR group (*p* < 0.001), indicating a transitional position between refusal and acceptance.

Knowledge and awareness barriers were also evident among the DLCR group. Participants who left the decision to their families perceived a greater lack of public awareness and education about organ donation in Kazakhstan compared to the LR group, while both groups scored lower than the LC group (*p* < 0.001).

Financial concerns also played a role in influencing preferences. The DLCR and LR groups reported significantly greater concerns about potential financial burdens associated with organ donation compared to the LC group (*p* < 0.001), though there was no significant difference between the LR and DLCR groups.

A critical finding was the high level of uncertainty about how to declare organ donation preferences among DLCR participants. Thus, DLCR participants reported significantly higher uncertainty than both the LR and the LC groups (*p* < 0.05).

### 3.2 Principal component analysis and group comparisons on barriers to organ donation

To identify the underlying dimensions of barriers to organ donation, a Principal Component Analysis (PCA) with Varimax rotation was conducted. The analysis extracted two components, accounting for 49.2% of the variance in the dataset. The first component, institutional and trust barriers (26.6% variance), encompassed concerns related to lack of trust in the medical system, ethical worries, financial concerns, and lack of procedural knowledge. Higher scores on this component indicated skepticism toward the healthcare system and organ donation procedures rather than personal or religious opposition. The second component, cultural and religious barriers (22.6% variance), captured deeply ingrained beliefs, including religious restrictions, discomfort with posthumous body use, and fears that willingness to donate could impact medical treatment decisions. These barriers reflect fundamental societal and psychological concerns that influence donation hesitancy.

**Figure 1:**
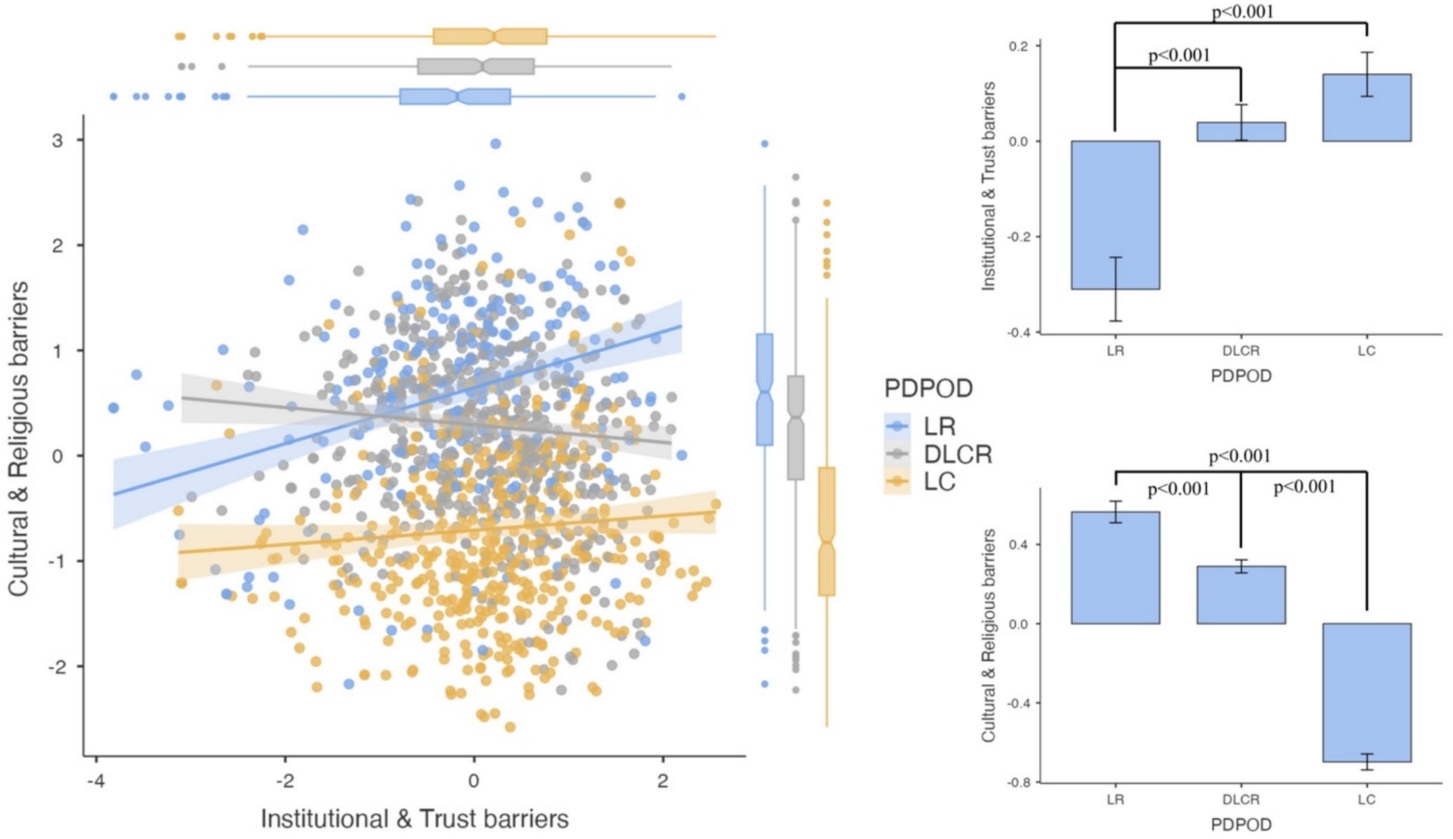
Comparison of institutional and trust barriers and cultural and religious barriers across posthumous organ donation decision groups. This figure presents PCA-extracted components of Institutional and Trust Barriers and Cultural and Religious Barriers across three donation decision groups: Lifetime Refusal (LR), Decision Left to Close Relatives (DLCR), and Lifetime Consent (LC). Left Panel: A scatterplot with regression lines shows the relationship between institutional and cultural barriers within each group. LR individuals (blue) exhibit the strongest positive association, while LC individuals (yellow) show the weakest. Top Right Panel: A bar plot compares Institutional and Trust Barrier Scores, showing significantly higher scores in LR, intermediate levels in DLCR, and the lowest in LC (*p* < 0.001). Bottom Right Panel: A bar plot illustrates Cultural and Religious Barrier Scores, where LR exhibits the highest, DLCR remains intermediate, and LC shows the lowest levels (*p* < 0.001).

Comparing these components across donation decision groups revealed significant differences (Figure 1). One-way ANOVA results showed that cultural barriers differed more strongly between groups than institutional barriers. Post-hoc tests indicated that LR individuals exhibited the highest levels of both institutional and cultural barriers, while LC individuals had the lowest. The DLCR group was statistically closer to LC in terms of institutional concerns (*p* = 0.227), but significantly aligned with LR in cultural barriers (*p* < 0.001). This suggests that institutional skepticism is not the primary factor preventing DLCR individuals from consenting to donation; rather, their hesitation stems from cultural and religious concerns.

Given these findings, intervention strategies should prioritize addressing cultural and religious hesitancy rather than focusing solely on institutional trust-building efforts. While lifetime refusers (LR) may require a combination of institutional transparency and cultural engagement, DLCR individuals could be influenced through faith-based discussions, personal narratives from donor families, and targeted educational campaigns to address body integrity concerns. The fact that DLCR individuals are institutionally similar to LC suggests that they may be more receptive to behavioral nudges or awareness initiatives that normalize lifetime consent. By focusing on cultural and emotional aspects rather than institutional skepticism, it may be possible to shift this undecided group toward proactive donor registration, thereby increasing organ donation rates.

#### 3.3 Attitudes toward posthumous organ donation among the DLCR group

Attitudes toward posthumous organ donation among participants who left the decision to their families (DLCR group) were mixed, reflecting a wide spectrum of perspectives. When asked whether they held a positive view of donating their organs after death, 9.0% (*n* = 52) of respondents in this group completely disagreed, while 15.7% (*n* = 91) disagreed, indicating that approximately one-quarter (24.7%) expressed negative attitudes toward organ donation. Meanwhile, 30.8% (*n* = 178) remained neutral, suggesting hesitation or uncertainty regarding their stance. In contrast, 30.6% (*n* = 177) agreed, and 13.8% (*n* = 80) completely agreed, meaning that 44.4% of the DLCR group had a favorable attitude toward organ donation.

#### 3.4 Predictors of posthumous organ donation attitudes among the DLCR group

To better understand the factors influencing attitudes toward posthumous organ donation among individuals who deferred the decision to their families (DLCR group), a linear regression analysis was conducted, with attitudes toward organ donation as the dependent variable. The overall model was significant (R^2^ = 0.304, *p* < 0.001), indicating that the included variables explained 30.4% of the variance in attitudes within this group (Table 4).

**Table 4:** Determinants of posthumous organ donation attitudes among individuals deferring the decision to family (DLCR group, *n* = 578).

Variable	Stand. Estimate	95% CI	*p*
Gender			
Female–Male	0.070	−0.117–0.260	0.463
Age	0.029	−0.070–0.128	0.566
Ethnicity			
Russian–Kazakh	−0.277	−0.662–0.108	0.158
Other–Kazakh	−0.147	−0.423–0.129	0.296
Language			
Russian–Kazakh	0.190	0.0001–0.380	0.050
Other–Kazakh	−0.027	−0.502–0.449	0.912
Occupation			
Employed–Student	−0.219	−0.517–0.079	0.149
Self-employed–Student	−0.025	−0.409–0.459	0.910
Unemployed–Student	0.179	−0.344–0.702	0.502
Pensioner–Student	0.326	−0.253–0.905	0.270
Educational level	0.062	−0.017–0.142	0.125
Specialization			
Medical–Non-medical	0.100	−0.095–0.295	0.312
Family status			
Married–Single	0.028	−0.302–0.359	0.867
Divorced–Single	−0.031	−0.424–0.362	0.876
Widow–Single	0.381	−0.136–0.898	0.148
Children			
Yes–No	−0.046	−0.385–0.294	0.792
Living area			
Urban–Rural	0.124	−0.052–0.31	0.167
Religion affiliation			
Christian–Islam	−0.013	−0.4082– 0.429	0.950
Agnosticism–Islam	0.411	0.070–0.753	0.018
Atheism–Islam	0.253	−0.092–0.597	0.150
Other–Islam	0.061	−0.745–0.868	0.881
Religiosity	0.063	−0.017–0.144	0.122
Economic well-being	−0.051	−0.124–0.021	0.166
Knowledge on organ donation	0.223	0.143–0.393	<0.001
Institutional and trust barriers	0.280	0.199–0.362	<0.001
Cultural and religious barriers	−0.290	−0.374–−0.207	<0.001

Most demographic factors did not significantly predict attitudes toward organ donation among individuals who deferred the decision to their families. Gender, age, ethnicity, educational level, medical specialization, family status, number of children, and economic well-being all showed no significant associations (*p* > 0.05). However, language preference had a marginal effect, with Russian-speaking participants demonstrating more favorable attitudes toward organ donation compared to Kazakh speakers (β = 0.190, *p* = 0.050). Additionally, agnostic participants exhibited significantly more positive attitudes toward organ donation than Muslims (β = 0.411, *p* = 0.018).

Knowledge about organ donation was a strong positive predictor of favorable attitudes (β = 0.223, *p* < 0.001), indicating that individuals with greater awareness were more inclined to support donation. Similarly, institutional and trust barriers were positively associated with donation attitudes (β = 0.280, *p* < 0.001), suggesting that those with higher concerns about procedural transparency and trust in the medical system may still hold favorable perceptions of organ donation.

In contrast, cultural and religious barriers emerged as the strongest negative predictor of donation attitudes (β = −0.290, *p* < 0.001), meaning that individuals with stronger cultural and religious concerns were significantly less likely to have positive views on organ donation. This aligns with previous findings that DLCR individuals are more aligned with Lifetime Refusal (LR) in cultural aspects, rather than with Lifetime Consent (LC).

Although specialization did not have a direct effect on attitudes toward posthumous organ donation in the regression analysis, we conducted a mediation analysis to examine whether institutional and trust barriers and cultural and religious barriers mediate the relationship between specialization (medical vs. non-medical background) and donation attitudes among individuals who deferred the decision to their families (Table 5).

**Table 5:** Mediation model (specialization: medical-non medical).

Type	Effect	Estimate	SE	*p*
Indirect	Specialization ⇒ Institutional and trust barriers ⇒ Attitudes	−0.128	0.042	<0.001
	Specialization ⇒ Cultural and religious barriers ⇒ Attitudes	−0.124	0.044	0.005
Component	Specialization ⇒ Institutional and trust barriers	−0.223	0.058	<0.001
	Institutional and trust barriers ⇒ Attitudes	0.666	0.077	<0.001
	Specialization ⇒ Cultural and religious barriers	0.194	0.066	0.003
	Cultural and religious barriers ⇒ Attitudes	−0.634	0.068	<0.001
Direct	Specialization ⇒ Attitudes	−0.029	0.103	0.777
Total	Specialization ⇒ Attitudes	−0.302	0.110	0.006

The analysis revealed that while specialization had no direct effect on attitudes (*p* = 0.777), it significantly influenced both institutional and trust barriers (β = −0.223, *p* < 0.001) and cultural and religious barriers (β = 0.194, *p* = 0.003). In turn, institutional and trust barriers were positively associated with donation attitudes (β = 0.666, *p* < 0.001), while cultural and religious barriers had a strong negative effect (β = −0.634, *p* < 0.001). The indirect effects of specialization on attitudes through these two mediators were both significant (*p* < 0.001 and *p* = 0.005, respectively), indicating that the relationship between specialization and donation attitudes operates entirely through these barriers.

These findings suggest that medical professionals experience lower institutional and trust barriers, which in turn positively influence their donation attitudes. However, they also report slightly higher cultural and religious barriers, which negatively impact their attitudes. This underscores the complexity of decision-making among medical professionals and highlights the need for targeted interventions that address both procedural trust and cultural concerns within this group.

## 4 Discussion

This study identified critical factors influencing individuals who defer posthumous organ donation decisions to their families (DLCR group), a substantial proportion (43.4%) among respondents in Kazakhstan. In line with previous work suggesting that undecided or ambivalent individuals are more amenable to persuasion than those who are firmly opposed (19), the DLCR group in our sample emerged as a crucial focal point for strategies designed to increase organ donor rates. Participants in the DLCR group demonstrated intermediate attitudes towards organ donation, significantly more positive than lifetime refusers (LR) but less favorable than those providing lifetime consent (LC). Importantly, individuals in the DLCR group expressed notably higher uncertainty about procedural aspects of declaring their donation status compared to other groups. Principal Component Analysis (PCA) further clarified that while institutional barriers, such as procedural concerns, played a role, cultural and religious barriers exerted a substantially stronger negative influence on organ donation attitudes in this group. Additionally, knowledge about organ donation emerged as a key positive predictor of favorable attitudes, suggesting targeted interventions addressing both informational deficits and cultural concerns could effectively shift undecided individuals towards proactive donor registration.

In our survey of 1,333 participants, we identified three distinct preference groups: those willing to donate their organs after death (35.3%), those refusing (21.4%), and a remarkably large undecided segment (43.4%) who said they would “leave the decision to their close relatives” (the DLCR group). The fact that nearly half of respondents in Kazakhstan prefer their family to decide indicates a strong cultural leaning toward collective decision-making. This aligns with Kazakhstan’s cultural and religious backdrop—the country has a Muslim-majority heritage and a post-Soviet communal ethos, both of which emphasize family and community in personal matters. Rather than making an autonomous declaration, many people feel it’s more appropriate or safer to let their next-of-kin choose at the time of death. Similar attitudes are seen in other societies with strong family orientation (20, 21). What is notable is that this group in Kazakhstan was not simply neutral or ignorant—they had identifiable attitudes and concerns that place them between the full consenters and refusers. The Kazakhstani DLCR respondents held moderately positive views on organ donation on average, significantly more favorable than those who outright refuse, yet not as positive as those who consent. In fact, 44.4% of the DLCR group expressed a favorable attitude toward donation in principle, suggesting that many are “persuadable” if their concerns are addressed. Their hesitation, therefore, is not due to lack of any altruistic feeling; it comes from unresolved barriers.

Demographically, individuals in the DLCR group in our study tended to be older, married or previously married, and parents, suggesting life-stage and family responsibilities significantly shape decision-making processes. This may be because individuals with familial obligations often defer decisions out of concern for the emotional and decisional burden on family members. Additionally, occupational status influenced decision preferences, with employed, pensioners, and unemployed participants more likely to defer, likely reflecting varying degrees of cultural barriers to organ donation decision-making (Supplementary Figure 1). Language preference and religious affiliation were also significant factors, with Kazakh-speaking individuals and those identifying as Muslim or Christian disproportionately represented in the DLCR group. This aligns with existing research from predominantly Muslim societies where religious beliefs about bodily integrity and posthumous practices heavily influence donation hesitancy (14). Thus, in predominantly Muslim countries, family and religious considerations are deeply intertwined in organ donation decisions. Strong family ties in Islamic communities mean people often defer to family opinion or worry about family objections. In Saudi Arabia, for instance, 15.3% of survey respondents who had not registered as donors cited anticipated family disapproval as a barrier, one of the top reasons alongside personal or religious concerns (22). Moreover, families themselves may feel uneasy with donation if they are unsure of religious permissibility or fear it will upset the mourning process. At the same time, muslim scholars from top academies have generally ruled that organ donation is permissible as an act of saving lives (23). These findings highlight the necessity of culturally sensitive educational initiatives to address these specific concerns.

Knowledge significantly predicted organ donation attitudes within the DLCR group, aligning with global literature underscoring the pivotal role of education in fostering favorable donation decisions (24, 25). Participants in the DLCR group exhibited intermediate levels of knowledge, lower than those expressing LC but similar to LR, suggesting that their hesitancy partly stems from insufficient awareness. Therefore, improved knowledge has been shown to positively correlate with willingness to donate, highlighting the transformative potential of targeted educational interventions.

Attitudes toward organ donation among DLCR individuals were notably intermediate between LC and LR groups, reflecting their ambivalence. Nearly half (44.4%) of the DLCR group reported favorable attitudes toward donation, suggesting that substantial segments of this group could be persuaded to consent if their informational needs are adequately addressed. Enhancing knowledge can reduce misconceptions and alleviate fears related to medical procedures and ethical practices, critical for converting their ambivalent attitudes into explicit consent.

A key finding from the study was that these family-deferers (DLCR) appear to be held back more by cultural/religious factors than by institutional ones. Through a factor analysis of barrier perceptions, we found DLCR individuals were institutionally similar to the willing donors (meaning they had relatively comparable trust in the system and knowledge levels), but culturally they aligned with those who refused (meaning they harbored similar concerns about religious or societal norms as the refusers). In other words, what separated this undecided group from becoming donors was not primarily procedural worries—it was deeper cultural and religious reservations.

Contrary to initial expectations that mistrust might directly deter organ donation, we observed a nuanced relationship between institutional concerns and attitudes within the DLCR group. While institutional skepticism, such as fears about unethical practices, trafficking, or reduced medical effort. Did correlate with overall hesitation, it also had a positive association with attitudes in the multivariable model. Thus, institutional barriers were not irrelevant among the Kazakhstani undecided group. In fact, one of the standout issues for DLCR individuals was confusion about the process of declaring one’s wishes. This is a clear institutional barrier—many did not know where or how to officially record their consent.

Cultural and religious objections stood out as the strongest negative predictors of donation attitudes among the DLCR group, aligning them more closely with lifetime refusers. This finding resonates with prior studies in predominantly religious or collectivistic contexts, where beliefs about body integrity and spiritual considerations shape end-of-life choices (26, 27). Notably, discomfort with posthumous body use and concerns about the religious legitimacy of donation were prevalent among DLCR participants, suggesting that culturally embedded values remain a formidable barrier to committed consent (28, 29). Given this reality, engaging with religious leaders and community influencers is vital. Faith-based endorsements and culturally congruent messages have been shown to ease anxieties rooted in religious doctrine, helping clarify that organ donation can be considered an act of altruism within many faith traditions (30).

### 4.1 Policy and practice implications

#### 4.1.1 Reducing procedural uncertainty

A key policy priority is to enhance public awareness of how individuals can officially record their posthumous organ donation decision using the existing e-government system in Kazakhstan. Although this digital infrastructure already enables people to register their consent or refusal online, our findings suggest that many remain uncertain about the precise legal and administrative steps. By demystifying the e-governmental registration process and reinforcing its security, policymakers can significantly reduce one of the most modifiable barriers to organ donation.

#### 4.1.2 Targeting cultural and religious concerns

Because cultural and religious barriers represent a potent deterrent, collaboration with community leaders, religious scholars, and grassroots organizations appears essential. Carefully designed public education campaigns and face-to-face community seminars could bridge the gap between the scientific rationale for organ donation and deeply ingrained cultural norms.

#### 4.1.3 Tailored engagement for medical professionals

Medical professionals in the DLCR group demonstrated an intriguing mix of high knowledge but also elevated cultural or religious reservations. Hospital-based initiatives that foster open dialogue about these concerns, host ethics workshops, and encourage peer support might be particularly effective. Such endeavors can underscore how professional expertise coexists with religious or cultural sensibilities and help clarify unresolved ethical dilemmas.

#### 4.1.4 Addressing language-based differences in attitudes

Language emerged as a subtle but important factor shaping attitudes toward posthumous organ donation. In our study, Russian-speaking participants were significantly more likely to hold favorable attitudes compared to Kazakh-speaking participants, even after adjusting for other demographic variables. This suggests that language is not merely a means of communication, but may also serve as a proxy for cultural framing, access to information, and exposure to different value systems. To bridge this attitudinal gap, public health campaigns, educational materials, and consent registration portals must be linguistically and culturally tailored. It is not enough to simply translate materials; content should be contextualized for cultural meaning, using different framings, messengers, and examples depending on the linguistic audience.

### 4.2 Strengths and limitations

A strength of this study is its large sample size, encompassing diverse sociodemographic groups across Kazakhstan. The use of principal component analysis (PCA), multinomial regression, and mediation models strengthened the analytical rigor and allowed for nuanced insights into how institutional versus cultural barriers influence decision-making about posthumous organ donation.

However, several important limitations must be acknowledged. First, the cross-sectional design prevents causal inference. Second, the study relied on an online survey, which likely excluded individuals with lower levels of education, limited digital access, or lower socioeconomic status. As a result, groups such as rural residents, older adults, and those with limited internet literacy may be underrepresented. This could partially explain why no significant difference was found between urban and rural groups, a contrast to findings in other countries. Third, the study sample was heavily skewed toward women, individuals with medical training, married participants, urban residents, and Muslims, each comprising around 80% of the total sample. This lack of representativeness limits the generalizability of the findings and may bias the associations observed. Future studies should adopt stratified or mixed-method recruitment strategies to better reach underrepresented groups and validate these findings across more diverse population segments.

## 5 Conclusion

This study’s findings underscore those individuals who defer their organ donation decisions (DLCR) are an essential population for targeted intervention. Although institutional uncertainties hinder proactive consent, cultural and religious concerns emerged as the more substantial barrier. By confronting these deeply held values through community engagement, providing explicit procedures for registration, and improving general knowledge about organ donation, there is considerable potential to transition large numbers of currently undecided individuals toward lifetime consent.

## Data Availability

The raw data supporting the conclusions of this article will be made available by the authors, without undue reservation.
