# Supplementary material for: Too uncertain to consent, too supportive to refuse: the sociocultural dilemma of hesitant organ donors in Kazakhstan
Source: Front Public Health. 2025 May 19;13:1602008. doi: 10.3389/fpubh.2025.1602008 (PMC12127333; doi:10.3389/fpubh.2025.1602008)
Supplement: Supplementary file 1 [file Data_Sheet_1.docx]

**Supplementary materials**

**Suppl. Table 1.** Adjusted Odds Ratios for the Effect of Language on Posthumous Organ Donation Preferences Across Sociodemographic Models

| Model | Covariate | Comparison | OR (Russian-Kazakh) | p | OR (Other-Kazakh) | p | R^2^_McF_ |
| --- | --- | --- | --- | --- | --- | --- | --- |
| 1 | Gender | DLCR-LR | 1.187 | 0.258 | 2.718 | 0.118 | 0.034 |
|  |  | LC-LR | 3.323 | <0.001 | 8.233 | <0.001 |  |
| 2 | Age | DLCR-LR | 1.175 | 0.258 | 2.608 | 0.134 | 0.067 |
|  |  | LC-LR | 3.303 | <0.001 | 7.271 | 0.002 |  |
| 3 | Occupation | DLCR-LR | 1.142 | 0.381 | 2.653 | 0.127 | 0.052 |
|  |  | LC-LR | 3.066 | <0.001 | 7.585 | 0.001 |  |
| 4 | Specialization | DLCR-LR | 1.171 | 0.309 | 2.659 | 0.126 | 0.036 |
|  |  | LC-LR | 2.960 | <0.001 | 7.651 | 0.001 |  |
| 5 | Family status | DLCR-LR | 1.162 | 0.325 | 2.647 | 0.128 | 0.047 |
|  |  | LC-LR | 2.973 | <0.001 | 7.659 | 0.001 |  |
| 6 | Children | DLCR-LR | 1.156 | 0.340 | 2.625 | 0.131 | 0.061 |
|  |  | LC-LR | 2.782 | <0.001 | 6.944 | 0.002 |  |
| 7 | Living area | DLCR-LR | 1.203 | 0.231 | 2.668 | 0.125 | 0.037 |
|  |  | LC-LR | 3.051 | <0.001 | 7.978 | <0.001 |  |
| 8 | Religion affiliation | DLCR-LR | 1.310 | 0.134 | 2.908 | 0.098 | 0.052 |
|  |  | LC-LR | 2.951 | <0.001 | 7.006 | 0.002 |  |
| 9 | Religiosity | DLCR-LR | 1.161 | 0.336 | 2.656 | 0.126 | 0.039 |
|  |  | LC-LR | 2.897 | <0.001 | 7.802 | 0.001 |  |
| 10 | Economic well-being | DLCR-LR | 1.212 | 0.205 | 2.682 | 0.123 | 0.034 |
|  |  | LC-LR | 3.385 | <0.001 | 8.081 | <0.001 |  |


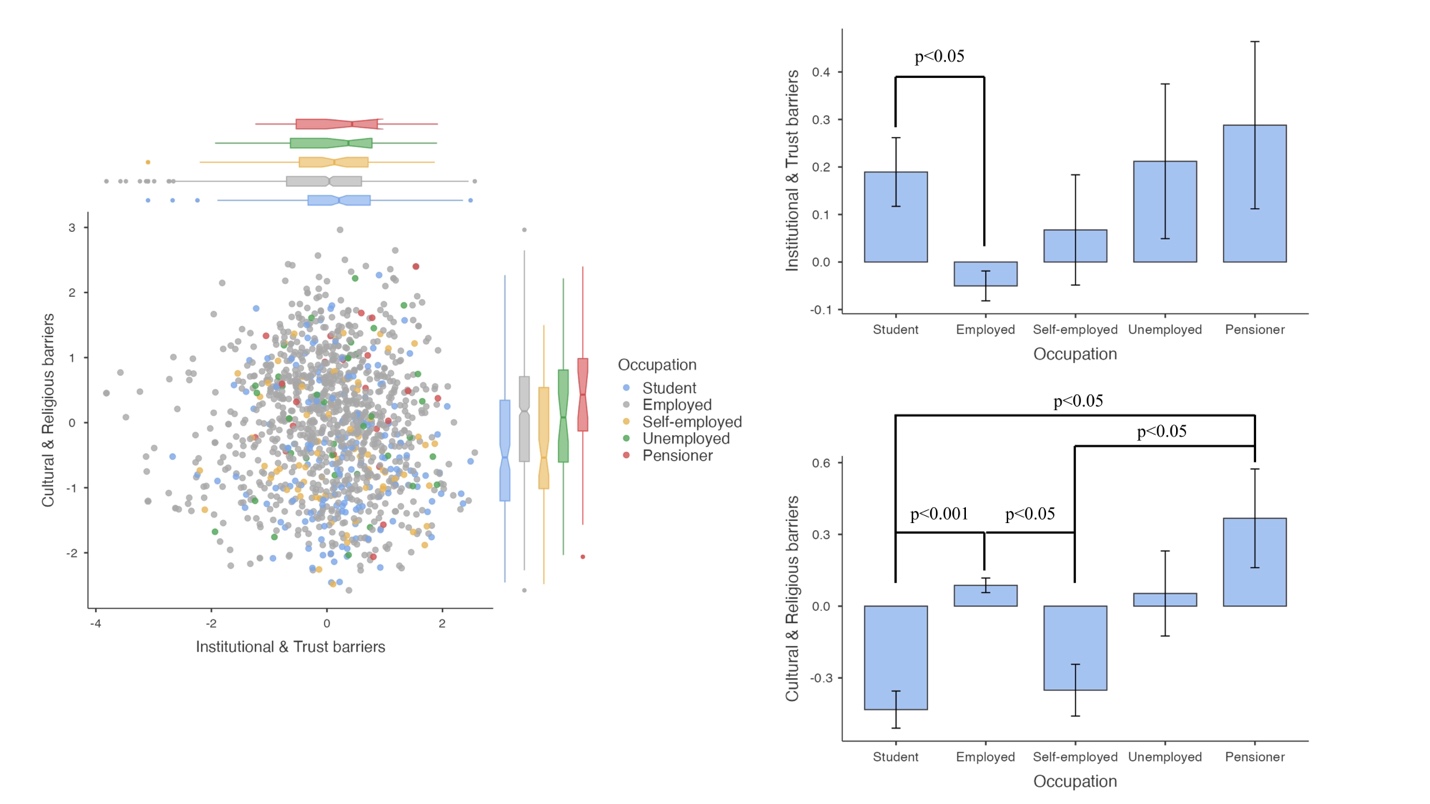


**Suppl. Figure 1.** Association Between Occupation and Reported Barriers to Posthumous Organ Donation

(Left): Scatter plot showing the distribution of participants by levels of Institutional & Trust Barriers (x-axis) and Cultural & Religious Barriers (y-axis), color-coded by occupation group. Marginal boxplots display the distribution of barrier scores by occupation. (Top right): Bar plot showing mean Institutional & Trust Barriers across occupational groups. Students reported significantly higher barrier levels than employed individuals (p < 0.05). (Bottom right): Bar plot showing mean Cultural & Religious Barriers across occupational groups. Students reported significantly lower levels of cultural and religious barriers compared to employed (p < 0.001), unemployed (p < 0.05), and pensioner groups (p < 0.05). Error bars represent standard errors of the mean. Pairwise comparisons were conducted using post hoc tests following ANOVA
